# Phylogenetic inferences of *Nepenthes* species in Peninsular Malaysia revealed by chloroplast (*trn*L intron) and nuclear (ITS) DNA sequences

**DOI:** 10.1186/s13104-017-2379-1

**Published:** 2017-01-26

**Authors:** Hamidun Bunawan, Choong Chee Yen, Salmah Yaakop, Normah Mohd Noor

**Affiliations:** 1 Institute of Systems Biology, Universiti Kebangsaan Malaysia (UKM), 43600 Bangi, Selangor Malaysia; 2 School of Environmental and Natural Resource Sciences, Faculty of Science and Technology, Universiti Kebangsaan Malaysia (UKM), 43600 Bangi, Selangor Malaysia

**Keywords:** ITS, Nepenthes, Peninsular Malaysia, Pitcher plant

## Abstract

**Background:**

The chloroplastic *trn*L intron and the nuclear internal transcribed spacer (ITS) region were sequenced for 11 *Nepenthes* species recorded in Peninsular Malaysia to examine their phylogenetic relationship and to evaluate the usage of *trn*L intron and ITS sequences for phylogenetic reconstruction of this genus.

**Results:**

Phylogeny reconstruction was carried out using neighbor-joining, maximum parsimony and Bayesian analyses. All the trees revealed two major clusters, a lowland group consisting of *N. ampullaria*, *N. mirabilis*, *N. gracilis* and *N. rafflesiana*, and another containing both intermediately distributed species (*N. albomarginata* and *N. benstonei*) and four highland species (*N. sanguinea*, *N. macfarlanei*, *N. ramispina* and *N. alba*).

**Conclusions:**

The *trn*L intron and ITS sequences proved to provide phylogenetic informative characters for deriving a phylogeny of *Nepenthes* species in Peninsular Malaysia. To our knowledge, this is the first molecular phylogenetic study of *Nepenthes* species occurring along an altitudinal gradient in Peninsular Malaysia.

## Background

Seven genera of pitcher plants namely *Cephalotus*, *Heliamphora*, *Darlingtonia, Brocchinia*, *Nepenthes*, *Sarracenia* and *Catopsis*. Pitcher plants have a worldwide distribution [1]. *Nepenthes* is the largest genus belonging to the family Nepenthaceae and consists of 120 species and five incompletely described taxa in seven geographical groupings: *Nepenthes* of Peninsular Malaysia and Indochina, *Nepenthes* of Borneo*, Nepenthes* of Sulawesi*, Nepenthes* of Sumatra and Java, *Nepenthes* of the Philippines, *Nepenthes* of New Guinea and Maluku Islands, and lastly *Nepenthes* of the Outlying Areas [1]. The endemic species of *Nepenthes* occur throughout Southeast Asia, particularly in the Sunda region, which includes Borneo, Sumatra, the Malay Peninsula, Java and some of the southern islands of the Philippines [2].

The *Nepenthes* flora in Peninsular Malaysia is relatively poor with only 11 species presently recorded (*N. alba* Ridl., *N. albomarginata* T. Lobb *ex* Lindl., *N. gracillima* Ridl., *N. ampullaria* Jack, *N. benstonei* C. Clarke, *N. gracilis* Korth., *N. macfarlanei* Hemsl., *N. rafflesiana* Jack, *N. mirabilis* (Lour.), Druce, *N. ramispina* Ridl. and *N. sanguinea* Lindl.). In comparison, there are 36 species recognized in Borneo, representing the greatest diversity of *Nepenthes* species in Southeast Asia [1, 3]. Clarke [2] reported only ten species of *Nepenthes* in Peninsular Malaysia, classifying *N. alba* as a heterotypic synonym of *N. gracillima*. Four of these species are restricted to montane habitats (*N. macfarlanei, N. gracillima, N. sanguinea, N. ramispina*), two species are found mainly on low hills (*N. albomarginata* and *N. benstonei*) and another four are lowland species (*N. ampullaria*, *N. gracilis*, *N. mirabilis* and *N. rafflesiana*). McPherson [1] described both *N. alba* and *N. gracillima* as distinct species.

The taxonomy of *Nepenthes* is based primarily on morphology (shape, color, size and ornamentation) [1–6]. Jebb and Cheek [7] recognized a single species, *N. vieillardii*, in New Caledonia into a single species, even though several species were recognized based on morphological variation. Kurata et al. [8] clarified the morphological diversity and verified the species classification of *N. vieillardii*, and they tentatively supported the taxonomic classification based on the pitcher morphology by Jebb and Cheek [7]. The current classification of *Nepenthes* in Peninsular Malaysia is also based on morphological characteristics, with distinct differences apparent between species found at high, intermediate and low altitudes.

Previous molecular phylogenetic studies of *Nepenthes* based on chloroplast (*trn*K intron and *mat*K gene) and nuclear (PRT1 and a translocated copy of *trn*K) sequences have provided a well-supported phylogeny of many species [9–11]. Using the plastid *trn*K intron in phylogenetic reconstruction, the three lineages can be separated according to habitat [9]. The first lineage included all species found in Sumatra, the Malay Peninsula and the Southeast mainland; the second lineage consisted of the species from Sulawesi, Borneo and the Philippines; and the third lineage comprised *Nepenthes* from New Guinea and Sulawesi [9, 11]. Meimberg and Heubl [11] also suggested that biogeographic “outlier species” occurring in Seychelles (*N. pervillei* Blume.), Sri Lanka (*N. distillatoria* L.), Madagascar (*N. madagascarensis* Poir. and *N. masoalensis* Schmid-Hollinger) and India (*N. khasiana* Hook. F.) are related to the three lineages consisting of all taxa from the Indo-Malay region.

Apart from molecular phylogenetic studies of *Nepenthes* using nuclear PRT and plastid *mat*K DNA sequences [9–11], there has been little development in the molecular systematics of *Nepenthes*. We report here the potential of the plastid *trn*L intron and nuclear ITS DNA sequences for the phylogenetic inference of *Nepenthes* in Malaysia. We also report here the phylogeographics of the *Nepenthes* species found in Peninsular Malaysia based on the DNA sequence data.

## Methods

### Sample collection

All the plant materials were collected from 11 sites (one individual from each of these localities) in Peninsular Malaysia, which covered nearly the complete natural range of the species except for *N. gracillima* Ridl (Table 1). Herbarium specimens were identified by Ruzi Rahman and deposited at the Universiti Kebangsaan Malaysia Herbarium (UKMB), Universiti Kebangsaan Malaysia.

**Table 1:** Sample location of *Nepenthes* species in Peninsular Malaysia and the outgroup species (*Sarracenia flava*)

Name	Accessions	State	Altitude (m)	GenBank (*trn*L intron)	GenBank (ITS)
*N. ampullaria*	INBIOSIS-N:AMP-2011	Johor–Ulu Tiram–Mersing	100–150	JX042566	JX042554
*N. gracilis*	INBIOSIS-N:GRA-2011	Selangor–Bukit Putri–UKM Bangi	100–150	JX042567	JX042555
*N. mirabilis*	INBIOSIS-N:MIR-2011	Selangor–Rawang/Batu Arang	50–100	JX042568	JX042556
*N. rafflesiana* var. *elongata*	INBIOSIS-N:RAF-E-2011	Johor–Ulu Tiram–Mersing	150–300	JX042569	JX042557
*N. rafflesiana* var. *nivea*	INBIOSIS-N:RAF-N-2011	Johor–Ulu Tiram–Mersing	150–300	JX042570	JX042558
*N. albomarginata*	INBIOSIS-N:ALBO-2011	Pahang–Cameron Highland	600–1300	JX042571	JX042559
*N. benstonei*	INBIOSIS-N:BEN-2011	Kelantan–Bukit Bakar	600–1000	JX042572	JX042560
*N. sanguinea*	INBIOSIS-N:SAN-2011	Pahang–Cameron Highland	1000–1500	JX042573	JX042561
*N. macfarlanei*	INBIOSIS-N:MAC-2011	Kelantan–Cameron Highland	800–1800	JX042574	JX042562
*N. ramispina*	INBIOSIS-N:RAM-2011	Pahang–Cameron Highland	600–1700	JX042575	JX042563
*N. alba*	INBIOSIS-N:ALBA-2011	Pahang–Cameron Highland	800–1800	JX042576	JX042564
*Sarracenia flava*	INBIOSIS-S:FLAVA-2011	Selangor	300 (*Sarracenia* originated from North America–grown in Selangor, lowland condition)	JX042577	JX042565

### Molecular procedures

DNeasy Plant Mini Kit (Qiagen, Germany) was used in extraction of total genomic DNA from fresh leaf tissue. The plastid *trn*L intron and the nuclear internal transcribed spacer (ITS) region consisting of ITS1, ITS2 and the 5.8S were PCR amplified. The amplification of the *trn*L intron was done using primers c (5′-CGA AAT CGG TAG ACG CTA CG-3′) and d (5′-GGG GAT AGA GGG ACT TGA AC-3′) [12], and the amplification of the ITS region was done using primers P17F (5′-CTA CCG ATT GAA TGG TCC GGT GAA-3′) and 26S-82R (5′-TCC CGG TTC GCT CGC CGT TAC TA-3′) [13]. A 100 µL PCR reaction was used in target gene amplification and consisted of the following components: 55 μL dH_2_O, 20 μL 10 × buffer, 10 μL of 25 mM MgCl_2_, 2.0 μL of 20 mM dNTPs, 1.0 μL each forward and reverse primers, 100 mM, 1.0 μL of *Taq* DNA polymerase (Promega) and 5 μL genomic DNA (20 ng/uL). The PCR was carried out in thermocycler (Applied Biosystem, USA) under the following cycling conditions: preliminary denaturation at 95 °C for 2 min; 35 cycles at 95 °C for 1 min, 45/66 °C for 1 min, and 72 °C for 1 min; final elongation at 72 °C for 5 min. The resultant PCR products were cleaned with PureLink™ PCR Purification Kit (Invitrogen) according to the manufacturer’s protocol.

ABI Prism Dye Terminator Cycle Sequencing Ready Reaction kit and ABI PRISM 3100 Genetic Analyzer (Perkin-Elmer, Foster City, CA) was used in DNA sequencing, following the manufacturer’s instructions. Direct sequencing was applied on both DNA regions in forward and reverse directions. The sequencing primers for the *trn*L intron were the same as the PCR primers. Primers P16F (5′-TCA CTG AAC CTT ATC ATT TAG AGG-3′) and P25R (5′-GGG TAG TCC CGC CTG ACC TG-3′) [13] were used to sequence the ITS region.

### Sequence analysis

ClustalX Multiple Sequence Alignment programme was used to align DNA sequences using the default settings. *Sarracenia flava* was designated as the outgroup in the phylogenetic analyses. The DNA sequences of the ITS region and *trn*L intron were combined in phylogenetic analyses. The evolutionary history was analyzed by neighbor-joining (NJ) and maximum parsimony (MP) methods using PAUP* 4.0b10 [14]. All positions containing gaps and missing data were removed from the dataset using the ‘complete deletion’ option. The pairwise distances generated using the uncorrected “p” model were used to construct the NJ tree. For the MP analysis, the cladograms were constructed utilizing unordered parsimony with equal weight. A heuristic search was conducted with the tree bisection–reconnection (TBR) branch swapping algorithm, random stepwise addition and ‘Mul-Trees’ option set on all the characters were weighted equally. The internal branch supports for the NJ and MP trees were assessed with Bootstrap analyses using 500 replicates. Bayesian inferences were carried out [15, 16] using MrBayes 3.12 [17]. The general reversible model with Gamma distributed rate heterogeneity (GTR + G) was selected by AIC using MrModelTest version 2.2 [18]. Two simultaneous metropolis-coupled Monte-Carlo Markov chains for >1,000,000 generations, a sample frequency of 100 generations and average standard deviation of split frequencies 0.009311 was used in Bayesian inference.

## Results

**Fig. 1:**
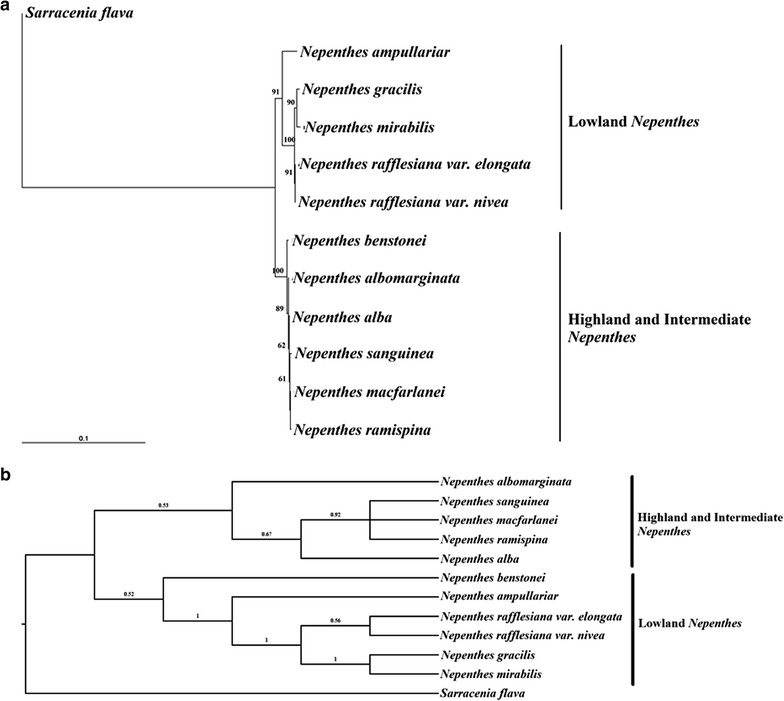
**a** Neighbor-joining tree generated based on the combined *trn*L and ITS sequence data with yellow pitcher plant (*Sarracenia flava*) as outgroup, a carnivorous plant native to the New World. The numbers above the branches represent the bootstrap percentage. **b** Majority-rule consensus tree resulted from the Bayesian analysis of *trn*L and ITS sequence data. Numbers at below branches are posterior probabilities. The bootstrap values from the MP analysis are shown above the branches

The PCR amplification and subsequent sequencing produced the ITS and *trn*L intron ITS fragments with size ranges of 555–559 and 673–705 bp, respectively. A total of 1311 characters were involved in the phylogenetic analyses of which 1012 were constant and 42 were parsimony-informative, while 257 variable characters were parsimony-uninformative. The pairwise genetic distances among the *Nepenthes* taxa ranged from 0 to 0.0342 (Table 2). The analyses of the combined *trn*L and ITS sequence data produced the NJ, MP and Bayesian trees with same topology. The NJ tree is shown in Fig. 1a. The MP analysis produced two most parsimonious trees with a tree length of 327 steps, CI = 0.9755 and RI = 0.9424. One of the parsimonious trees was similar to the topology of the Bayesian inference tree (Fig. 1b). The bootstrap values obtained from the MP analysis are shown above the branches.

**Table 2:** Pairwise distances of *Nepenthes* species generated from the combined *trn*L intron and ITS sequences based on uncorrected “p” model

	1	2	3	4	5	6	7	8	9	10	11	12
*N. gracilis*	–											
*N. rafflesiana* var*. elongata*	0.00405	–										
*N. ampullaria*	0.02427	0.02346	–									
*N. mirabilis*	0.00488	0.00569	0.02522	–								
*N. rafflesiana* var. *nivea*	0.00405	0.00000	0.02346	0.00569	–							
*N. albomarginata*	0.03078	0.02673	0.02876	0.03169	0.02673	–						
*N. benstonei*	0.03079	0.02673	0.02796	0.0317	0.02673	0.00238	–					
*N. sanguinea*	0.03324	0.02919	0.03119	0.03415	0.02919	0.00318	0.00477	–				
*N. macfarlan*ei	0.03243	0.02837	0.03038	0.03333	0.02837	0.00159	0.00397	0.00158	–			
*N. ramispina*	0.03324	0.02919	0.03119	0.03415	0.02919	0.00238	0.00477	0.00237	0.00079	–		
*N. alba*	0.03159	0.02753	0.02955	0.03249	0.02753	0.00079	0.00317	0.00238	0.00079	0.00159	–	
*Sarracenia flava*	0.2265	0.22316	0.22292	0.22296	0.22316	0.21805	0.21710	0.2185	0.21783	0.21783	0.21721	–

All the trees indicated the formation of two major genetic lineages: (1) Clade A (*N. albomarginata*, *N. benstonei, N. sanguinea*, *N. macfarlanei, N. ramispina* and *N. alba)* and (2) Clade B (*N. ampullaria*, *N. mirabilis*, *N. rafflesiana* and *N. gracilis).* These two major clades were supported by high bootstrap values and good posterior probability values (Fig. 1).

## Discussion

According to Clarke [2], only ten species of *Nepenthes* are presently recognized in Peninsular Malaysia on the basis of morphology. Four of these species are confined to montane habitats, two are found primarily on low hills and the remainders are lowland species that are common throughout the Sunda region. Based the combined *trn*L and ITS data, the lowland *Nepenthes* taxa in Peninsular Malaysia (*N. ampullaria*, *N. rafflesiana, N. mirabilis* and *N. gracilis*) clustered together to form a clade. On the other hand, the two *Nepenthes* species found on the low hills (*N. albomarginata* and *N. benstonei*) and four highland species (*N. sanguine*, *N. macfarlanei, N. ramispina* and *N. alba*) formed another clade. Therefore, this format of clustering corresponds to the altitudinal features of the natural growing habitats of *Nepenthes* in Peninsular Malaysia, suggesting differences in altitudinal growing environments probably played an important role in driving species radiation in the genus [19].

The four lowland *Nepenthes* species (*N. ampullaria*, *N. rafflesiana, N. mirabilis* and *N. gracilis)* that occur in Peninsular Malaysia are the same species that form the lowland suite in Sumatra. Two species are primarily found at intermediate altitudes—*N. albomarginata* and *N. benstonei* in both Malaysia and Sumatra. Of the four highland species, two (*N. sanguinea* and *N. macfarlanei*) have relatively wide distributions, whereas the other two (*N. ramispina* and *N. gracillima*) are more restricted. *N. albomarginata* is sister to one of the major clades, whereas *N. benstonei* is basal to this entire group of taxa (Fig. 1). This may imply that the highland species have most likely evolved from the lowland species with the transitional form of intermediate altitude species. Long term isolation of each population and limited seed flow, demonstrated in *Nepenthes vieillardii* of New Caledonia [20], might partly explain the speciation of highland *Nepenthes* species of Peninsular Malaysia. Climate may also play a role in the speciation of *Nepenthes,* with changes in vegetation, soil type and nutrient availability permitting a range of distinct ecological niches for *Nepenthes* to exploit.

Phylogenetic relationships in several carnivorous genera have been inferred using ITS sequences. Use of nrITS2 and nrITS1 in phylogeny reconstruction of 29 species of *Pinguicula* showed that the molecular phylogeny was congruent with the morphological classification [21].

## Conclusions

The *trn*L intron and ITS sequence data utilised in this work provided informative characters for the molecular phylogenetic inference of *Nepenthes* species in Peninsular Malaysia. The *Nepenthes* of Peninsular Malaysia formed two major clusters according to altitudinal distribution. It is reasonable to assume that the highland species have evolved from the lowland species. To our knowledge, this is the first attempt of applying the ITS and *trn*L intron sequence data as potential markers for *Nepenthes* species in Peninsular Malaysia. Worldwide, there are over 120 described species of *Nepenthes* and future work could consider the relationship between these species and those found in Peninsular Malaysia.

## Abbreviations

ITS: internal transcribed spacer
UKMB: Universiti Kebangsaan Malaysia Herbarium
NJ: neighbor-joining
MP: maximum parsimony
